# Supplementary material for: Cutting-Edge Strategies for Renal Tumour-like Lesions in Granulomatosis with Polyangiitis: A Systematic Review
Source: Diagnostics (Basel). 2024 Mar 6;14(5):566. doi: 10.3390/diagnostics14050566 (PMC10930867; doi:10.3390/diagnostics14050566)
Supplement: Supplementary file 1 [file diagnostics-14-00566-s001.zip › Supplementary Table S1.pdf]

Supplementary Table S1. Risk of bias scores of the included studies according to the Murad's tool [8].

Author	Selection	Ascertainment	Causality	Reporting		Quality of the study
	Does the patient(s) represent(s) the whole experience?	Was the diagnosis accurately established?	Were other significant diagnoses ruled out?	Were all crucial data referenced in the report?	Was the outcome correctly determined?	
**Tiwari et al [10]**	N	Y	Y	Y	Y	Moderate
**Maguire et al [11]**	N	Y	Y	N	Y	Low
**Schapira et al [12]**	N	Y	Y	N	Y	Low
**Schydlowsky et al [13]**	N	Y	Y	Y	Y	Moderate
**Smith et al [14]**	N	Y	Y	N	Y	Low
**Boubenider et al [15]**	N	Y	Y	Y	Y	Moderate
**Fairbanks et al [16]**	N	Y	Y	Y	Y	Moderate
**Dufour et al [17]**	N	Y	N	Y	Y	Low
**Thomas et al [18]**	N	Y	Y	N	Y	Low
**Verswijvel et al [19]**	N	Y	Y	Y	Y	Moderate
**Carazo et al [20]**	N	Y	Y	Y	Y	Moderate
**Kapoor et al [21]**	N	Y	Y	Y	Y	Moderate
**Leung et al [22]**	N	Y	Y	Y	Y	Moderate
**D'Hauwe et al [23]**	N	Y	Y	Y	Y	Moderate
**Krambeck et al [24]**	N	Y	Y	Y	Y	Moderate
**Vandergheynst et al [25]**	N	Y	Y	Y	Y	Moderate
**Vandergheynst et al [26]**	N	Y	Y	Y	Y	Moderate
**Sichani et al [27]**	N	Y	Y	Y	Y	Moderate
**Negi et al [28]**	N	Y	N	Y	Y	Low
**Lo Gullo et al [29]**	N	Y	Y	Y	Y	Moderate
**Frigui et al [30]**	N	Y	Y	Y	Y	Moderate
**Xu et al [31]**	N	Y	Y	Y	Y	Moderate
**Roussou et al [32]**	N	Y	Y	Y	Y	Moderate

**Ahmed et al [33]**	N	Y	Y	Y	Y	Moderate
**Ward et al [34]**	N	Y	Y	N	Y	Low
**Yamamoto et al [35]**	N	Y	Y	Y	Y	Moderate
**Fu et al [36]**	N	Y	Y	Y	Y	Moderate
**Higashihara et al [37]**	N	Y	Y	Y	Y	Moderate
**Dai et al [38]**	N	Y	Y	Y	Y	Moderate
**Guo et al [39]**	N	Y	Y	Y	Y	Moderate
**Kumar et al [40]**	N	Y	Y	Y	Y	Moderate
**Reeders et al [41]**	N	Y	Y	Y	Y	Moderate
**Villa-Forte et al [42]**	N	N	Y	N	Y	Low
**Boncoraglio et al [43]**	N	Y	Y	Y	Y	Moderate
**Abudaff et al [44]**	N	Y	Y	Y	Y	Moderate
**Bicakcigil et al [45]**	N	Y	Y	N	Y	Low
**Gregorini et al [46]**	N	Y	Y	Y	Y	Moderate
**Kaikoi et al [47]**	N	Y	Y	Y	Y	Moderate
**Nketiah Sarpong et al [48]**	N	N	Y	Y	Y	Low
**Ramasamy et al [49]**	N	N	Y	N	Y	Low
**Varkala S. et al [50]**	N	Y	Y	Y	Y	Moderate

The quality of the studies was assessed using the Murad's tool [8] for case series and case reports. Each query received a score of 1 (indicating yes) or 0 (indicating no). The study's quality was categorized as high, moderate, or low based on total scores of 5, 4, or ≤ 3, respectively.
Y: yes; N: no.
