# Supplementary material for: Cutting-Edge Strategies for Renal Tumour-like Lesions in Granulomatosis with Polyangiitis: A Systematic Review
Source: Diagnostics (Basel). 2024 Mar 6;14(5):566. doi: 10.3390/diagnostics14050566 (PMC10930867; doi:10.3390/diagnostics14050566)
Supplement: Supplementary file 1 [file diagnostics-14-00566-s001.zip › Supplementary Table S2.pdf]

Supplementary Table S2. Complete clinical, histological and radiological features of the 42 published GPA patients presenting with renal masses and our case.

Author	Sex / Age (y)	Extra-renal organ involvement	CRP (mg/L) ESR (mm/h)	ANCA	Kidney tests	Imaging	Localization	Biopsy	Kidney removal	Renal histology				Exclusion of other cause of granuloma	Steroids	Induction treatment	Other treatment	Outcomes	Follow-up imaging
										GN	Granuloma	Vasculitis	Fibrosis						
**Tiwari et al [10]**	F, 60	ENT, ocular	NA / 85	Anti-MPO (>750 U/mL)	Normal	US, CECT, PET	Unilateral	Yes	No	-	+	-	-	+	PN (60mg/d)	MTX (20mg/w)	-	+	US
**Maguire et al [11]**	F, 27	ENT, pulmonary	NA	Anti-PR3	NA	Renal arteriography	Unilateral	NA	Partial	+	+	-	+	NA	NA	CYC	NA	+	NA
**Schapira et al [12]**	M, 45	ENT, pulmonary	NA / 140	NA	Proteinuria	Gallium scan, US, angiography	Unilateral	No	Partial	+	+	-	-	+	PN	CYC	NA	+	NA
**Schydlowsky et al [13]**	M, 47	ENT, pulmonary	NA / 101	ANCA	Normal	US, technetium scintigrams, CECT	Unilateral	No	Radical	+	+	+	-	NA	PN (50 mg/d)	CYC (oral)	NA	+	NA
**Smith et al [14]**	F, 52	ocular	NA / normal	NA	Sterile pyuria	US, CT	Unilateral	No	Radical	+	+	-	-	NA	PN	AZA	-	+	NA
**Boubenider et al [15]**	F, 45	ENT, skin	NA / 91	c-ANCA (37 EU)	Renal failure, haematuria, proteinuria	US, CT	Unilateral	Yes	Radical	+	+	+	+	+	-	-	-	ESRD (hemodialysis)	NA
**Fairbanks et al [16]**	M, 68	ENT, pulmonary	NA – 115	p-ANCA 1:20	Normal	CECT	Unilateral	Yes	No	+	+	+	-	NA	PN (40mg/d)	MTX (15mg/w)	-	+	CT

**Dufour et al [17]**	M, 70	ENT, neurologic, pulmonary	NA	Anti-MPO	Renal failure	CT	Unilateral	No	Radical	+	+	+	-	NA	MPN	CYC	-	+ (2 relapses)	NA
**Dufour et al [17]**	M, 67	MSK, pulmonary, CNS	350 / NA	Anti-PR3 (320)	Renal failure	CT	Unilateral	NA	No	NA	NA	NA	NA	NA	MPN	CYC	-	- (death due to severe CNS involvment)	CT (renal masses decreased in size)
**Thomas et al [18]**	F, 48	ENT, PNS, ocular	NA / 106	NA	Renal failure	IVU, CT	Unilateral	Yes	Radical	+	+	+	+	NA	PN	CYC	-	+	-
**Verswijvel et al [19]**	M, 24	ENT, spleen	NA	Anti-PR3 (151 AU)	Renal failure, haematuria	US, CECT, MRI	Unilateral	Yes	No	+	-	-	NA	NA	MPN	CYC	-	+	US
**Carazo et al [20]**	M, 29	ocular	NA	Anti-PR3	Normal	US, IVU, CECT	Bilateral	Yes	Radical	+	+	+	+	NA	PN	CYC	-	+ (1 ocular relapse)	CT
**Kapoor et al [21]**	M, 22	-	NA	Anti-PR3	Renal failure, haematuria, proteinuria	US, MRI	Bilateral	Yes	No	+	-	+	-	+	-	-	-	ESRD (hemodialysis)	NA
**Leung et al [22]**	M, 66	ENT, skin	109 / 76	Anti-MPO	Normal	US, CT	Bilateral	Yes	No	-	+	-	+	+	PN (60mg/d)	MTX (oral 25mg/w)	-	+	CT
**D'Hauwe et al [23]**	F, 14	ENT, MSK	72 / NA	-	Pyuria	US, CECT	Unilateral	Yes	No	-	+	-	-	+	PN	RTX (4 pulses) + MTX	-	+	US
**Krambeck et al [24]**	M, 61	ENT, CNS	+ / +	-	Normal	CECT	Unilateral	No	Partial	-	+	-	-	+	PN (40mg/d)	AZA (150mgx2)	-	+	NA

**Vanderghеynst et al** [25]	M, 32	PNS	335 / 78	Anti-PR3	Proteinuria	CT	Unilateral	Yes	Partial	+	+	-	+	+	MPN pulses (1g/d)	CYC (0.7 g/m^2)	-	+	NA
**Vanderghеynst et al** [26]	F, 23	ENT, skin, endocrine system	110 / NA	Anti-MPO	Normal	PET, CEUS	Bilateral	Yes	No	+	+	-	+	NA	PN	RTX (4 pulses)	NA	+	US,
**Sichani et al** [27]	F, 22	ENT, pulmonary, DAH	136/ +	ANCA	Proteinuria,haematuria	US, CECT	Unilateral	Yes	No	-	+	+	-	+	-	-	-	- (death before diagnosis and treatment)	NA
**Negi et al** [28]	M, 40	ENT	142 → 320 / 101 → 124	Anti-PR3 (1:320)	Renal failure	CT	Bilateral	No	No	NA	NA	NA	NA	+	NA	NA	-	+	CT
**Lo Gullo et al** [29]	M, 38	ENT, pulmonary	124 / eleveted	Anti-PR3 (49 UI/mL)	Normal	US, CECT, PET	Unilateral	Yes	No	-	+	-	-	+	PN (48mg/d)	RTX (1gx2)	-	+	CT
**Frigui et al** [30]	F, 59	ENT, ocular	202 / 135	Anti-PR3	Proteinuria	US, CECT	Bilateral	Yes	No	-	+	+	+	+	MPN pulses (1g/d)	CYC iv	MTX (20mg/w)	+	CT
**Xu et al** [31]	M, 55	-	NA	Anti-PR3 (206 RU)	Haematuria	CT	Unilateral	No	Radical	+	-	+	+	NA	PN	NA	NA	+	NA
**Roussou et al** [32]	F, 72	ENT	171 / 90	p-ANCA (1:20)	Normal	CECT, MRI	Unilateral	No	Radical	+	+	-	NA	NA	PN (40mg/d)	CYC (2mg/Kg)	-	+	NA
**Ahmed et al** [33]	F, 28	Pulmonary	187 / 83	Anti-PR3 (>250 AU/mL)	Haematuria	US, CT	Bilateral	Yes	No	+	+	-	+	+	PN	CYC iv (5.1g)	NA	+	US

**Ward et al** [34]	F, 48	ENT, CNS, pulmonary	+ / +	c-ANCA (1:20)	NA	CT	Unilateral	Yes	Radical	+	+	-	+	+	MPN pulses	CYC oral	MPN pulses + RTX (relapse)	- (CYC) + (RTX)	-
**Yamamoto et al** [35]	M, 60	-	24 / NA	Anti-MPO (10.4 U/mL)	Normal	CE - CT MRI	Unilateral	Yes	Radical	-	+	+	+	+	PN (30mg/d)	-	-	+	-
**Fu et al** [36]	F, 33	ENT	86 / 42	-	Normal	US, PET	Bilateral	Yes	No	+	+	NA	NA	NA	PN	CYC	NA	+	NA
**Higashihara et al** [37]	F, 75	-	70 / NA	-	Renal failure	CECT	Unilateral	No	Radical	+	+	+	+	+	PN (30mg/d)	-	-	+	-
**Dai et al** [38]	M, 32	ENT, pulmonary	NA	Anti-PR3 (8.6 U/mL)	Normal	MRI, PET	Unilateral	Yes	No	-	+	-	+	+	MPN	CYC	-	+	CT
**Guo et al** [39]	F, 71	Pulmonary, ocular	102.9 /109	Anti-PR3 (6.99 U/ml)	Normal	CT	Bilateral	Yes	No	-	+	-	+	+	PN (40 mg/d)	RTX (1gx2)	RTX	+	CT
**Kumar et al** [40]	F, 27	ENT	127 / 57	Anti-PR3	Normal	US, CECT, IVU	Unilateral	Yes	No	-	+	-	-	+	PN (60 mg/d)	RTX (AZA intolerance)	-	+	CT
**Reeders et al** [41]	M, 46	Pulmonary, skin, articular	100 / NA	Anti -PR3 (36 U/mL)	Stable renal failure	CT	Unilateral	No	Radical	-	+	+	-	+	MPN (80mg/d)	RTX (4 pulses) + CYC (1 dose)	-	+	-
**Villa-Forte et al** [42]	M, 45	ENT, pulmonary	NA	NA	NA	NA	Unilateral	No	Radical	+	+	+	NA	+	PN	CYC	-	ESRD (hemodialysis)	NA

**Boncoraglio et al** [43]	M, 47	ENT	+ / +	Anti- PR3	Renal failure, haematuria, proteinuria	US, CT, MRI	Unilateral	No	Partial	+	+	-	+	NA	PN (1mg/kg/d)	RTX (1gx2)	RTX	+	NA
**Abudaff et al** [44]	M, 20	ENT	173 / 140	Anti-PR3	Renal failure, haematuria, proteinuria,	US, CT	Unilateral	Yes	No	+	NA	+	NA	NA	PN	RTX	-	+	NA
**Bicakcigil et al** [45]	F, 47	ENT, breast, spleen	NA	NA	NA	CT	Bilateral	Yes	No	NA	NA	NA	NA	NA	MPN	CYC	-	+	CT
**Gregorini et al** [46]	M, 46	ENT, CNS	NA	Anti-PR3 (186 RU/mL)	Normal	CT	Unilateral	Yes	No	-	+	-	-	+	PN (1mg/kG/d)	RTX (375mg/m2) + CYC (2mg/Kg/d)	-	+	CT
**Kaikoi et al** [47]	F, 76	ENT, ocular,	297 / NA	Anti PR3 (271.7 U/mL)	Minimal proteinuria	CECT	Unilateral	Yes	No	+	+	+	-	NA	MPN pulses	CYC	NA	+	CT
**Nketiah Sarpong et al** [48]	M, 57	Pulmonary, DAH	NA	Anti-PR3	Renal failure, haematuria, proteinuria	NA	Unilateral	Yes	No	+	+	+	-	+	MPN	RTX	NA	+	NA
**Ramasamy et al** [49]	F, 39	NA	NA	-	Renal failure	NA	Unilateral	No	Radical	+	+	+	-	NA	NA	NA	NA	ESRD	NA
**Varkala S. et al** [50]	M, 22	Pulmonary, DAH, endocarditis, skin	NA	Anti-PR3 (>100)	Renal failure, haematuria, leukocyturia, granular casts	CECT	Unilateral	Yes	No	+	-	-	-	+	MPN	CYC	NA	+	CECT

**Present case**	F, 49	-	160/ 120	Anti-PR3 (42.3 KU/L)	Normal	US, CECT, PET-MRI	Bilateral	Yes	No	-	+	-	-	+	MPN	RTX	-	+	US, CECT

ANCA antineutrophil cytoplasmic antibodies, AZA azathioprine, cANCA cytoplasmatic-ANCA, CE contrast enhanced, CEUS contrast-enhanced ultrasonography, CNS central nervous system, CRP c-reactive protein, CT computer tomography, CYC cyclophosphamide, DAH diffuse alveolar haemorrhage, ENT ear nose and throat, ESRD end stage renal disease, ESR erythrocyte sedimentation rate, IV intravenous, IVU intravenous urography, MPN methylprednisolone, MPO myeloperoxidase, MRI magnetic resonance imaging, MTX methotrexate, NA not available, pANCA perinuclear-ANCA, PET positron-emission tomography, PN prednisolone, PNS peripheral nervous system, PR3 proteinase 3, RTX rituximab, US ultrasound.
